# Six adenoviral vectored African swine fever virus genes protect against fatal disease caused by genotype I challenge

**DOI:** 10.1128/jvi.00622-24

**Published:** 2024-07-02

**Authors:** Raquel Portugal, Hannah Goldswain, Rebecca Moore, Matthew Tully, Katie Harris, Amanda Corla, John Flannery, Linda K. Dixon, Christopher L. Netherton

**Affiliations:** 1 The Pirbright Institute, Woking, Surrey, United Kingdom; Lerner Research Institute, Cleveland Clinic, Cleveland, Ohio, USA

**Keywords:** African swine fever, vaccines, adenovirus vectors, viral hemorrhagic fever, pig

## Abstract

African swine fever virus causes a lethal hemorrhagic disease in domestic swine and wild boar for which currently licensed commercial vaccines are only available in Vietnam. Development of subunit vaccines is complicated by the lack of information on protective antigens as well as suitable delivery systems. Our previous work showed that a pool of eight African swine fever virus genes vectored using an adenovirus prime and modified vaccinia virus boost could prevent fatal disease after challenge with a virulent genotype I isolate of the virus. Here, we identify antigens within this pool of eight that are essential for the observed protection and demonstrate that adenovirus-prime followed by adenovirus-boost can also induce protective immune responses against genotype I African swine fever virus. Immunization with a pool of adenoviruses expressing individual African swine fever virus genes partially tailored to genotype II virus did not protect against challenge with genotype II Georgia 2007/1 strain, suggesting that different antigens may be required to induce cross-protection for genetically distinct viruses.

**IMPORTANCE:**

African swine fever virus causes a lethal hemorrhagic disease in domestic pigs and has killed millions of animals across Europe and Asia since 2007. Development of safe and effective subunit vaccines against African swine fever has been problematic due to the complexity of the virus and a poor understanding of protective immunity. In a previous study, we demonstrated that a complex combination of eight different virus genes delivered using two different viral vector vaccine platforms protected domestic pigs from fatal disease. In this study, we show that three of the eight genes are required for protection and that one viral vector is sufficient, significantly reducing the complexity of the vaccine. Unfortunately, this combination did not protect against the current outbreak strain of African swine fever virus, suggesting that more work to identify immunogenic and protective viral proteins is required to develop a truly effective African swine fever vaccine.

## INTRODUCTION

African swine fever virus (ASFV) is the etiological agent of African swine fever (ASF), a disease of domestic pigs and wild boar, which has been reported in Africa, Asia, Europe, North America, and Oceania since 2018. Disease typically manifests as an acute hemorrhagic fever with death occurring within 4 to 10 days after exposure. Transmission is primarily through direct contact with infected animals and fomites or ingestion of uncooked pork products. In East Africa, the soft tick vectors *Ornithodoros moubata or porcinus* can act as a biological vector and maintain a reservoir in the sylvatic cycle involving warthogs. Currently, there are no widely available commercially licensed vaccines against ASFV, and therefore, control is predicated on strict biosecurity on the farms, rapid diagnosis of affected animals followed by quarantine and culling of herds. ASFV can be classified into different genotypes (I–XXIV) based on partial sequencing of the *B646L* gene (1) that encodes for the major capsid protein. The current ASF panzootic in Europe and Asia is caused by a genotype II virus. A number of modified live virus (MLV) vaccine candidates have been developed against ASFV in recent years with promising safety profiles (2–5), and progress has been made in identifying cell lines suitable for propagating such vaccines (6–8). Concerns still remain about reversion to virulence (3, 9), and a differentiating infected from vaccinated animals (DIVA) candidate is not yet available (10, 11).

As inactivated ASFV does not protect pigs (12–14), a subunit vaccine would alleviate the safety concerns associated with the use of MLVs by avoiding the use of the live virus entirely and could be DIVA-compliant by design. The complexity of the virus and our poor understanding of the mechanisms of protective immunity have made the identification of protective antigens challenging. Combinations of the *CP204L*, *E183L*, and *EP402R* genes expressed as fusions either by baculovirus (15–18) or by plasmids (19) protect a proportion of pigs from genotype I challenge, and a DNA library vaccination approach was similarly successful (20). Different immunization regimes with these three genes induce different immune responses, providing evidence for the importance of both the antibody response (15, 16, 18) and T-cell response in protection (17, 19). Both the antibody and T-cell responses play a role in the protection derived from live-attenuated viruses’ (LAV) immunization as passive transfer of sera from recovered animals can protect animals from both homologous and heterologous challenges (21, 22) and depletion of CD8 cells abrogates protection (23).

Viral vectors expressing individual ASFV proteins can induce robust antigen-specific immune responses, and we have demonstrated that a combination of the *B602L*, *B646L*, *CP204L*, *E183L*, *E199L*, *EP153R*, *F317L,* and *MGF505-5R* genes delivered through a replication-deficient human adenovirus 5 (rAd) prime followed by Modified Vaccinia Ankara (MVA) boost was sufficient to protect pigs from challenge with the virulent genotype I isolate, OUR T1988/1 (24). Although immune responses against all eight proteins were evident, it is unlikely that all of them were required for protection and progress toward a viral vectored vaccine against ASF requires rationalization of the antigens required for protection. Similarly, further modification of the pool of antigens or the immune regime will be needed to reduce clinical signs and levels of viremia in immunized animals after challenge. Efforts to induce protective immunity using subunit vaccines against genotype II virus have so far been unsuccessful (25–29), although reports of the combinations of antigens and delivery systems that were successful against genotype I being tested in genotype II challenge models are lacking. Therefore, we aimed to test different combinations of viral vectors (rAd and MVA) expressing protective antigens in immunization and challenge experiments, as well as examine the protection of effective combinations against genotype II challenge.

## RESULTS

### Immune responses induced by different combinations of rAd expressing genotype I ASFV genes

A pool of eight antigens delivered using a recombinant adenovirus (rAd) prime and modified vaccinia Ankara (MVA) boost protected pigs from severe disease caused by a genotype I isolate of ASFV (24). The next question was whether we could refine the immunization regime by reducing the number of antigens in the pool or replacing the rAd/MVA heterologous prime-boost with rAd homologous prime-boost. Groups of five pigs were immunized with pools of rAd via the intramuscular route and then boosted 4 weeks later with either pools of MVA or adenovirus (Table 1). Six weeks after boost, the animals were challenged with OUR T1988/1, a virulent genotype I ASFV isolate. Antigen pools A and D were delivered using rAd-prime/MVA-boost, with the exception of *MGF505-*5R, which was vectored using rAd alone. Animals in pools, B, C, and E were immunized using rAd-prime and boost. Antigen pool A comprised the eight antigens shown to protect animals from severe disease and antigen pool D comprised *B602L*, *E183L*, and *EP153R* as removing these three antigens from antigen pool A abrogated the protective immune response (24). The other three pools comprised different combinations of the eight antigens (Table 1).

**TABLE 1:** Composition of antigen pools in experiments 1 and 2^*a*^

Experiment	1					2	
Pool	A	B	C	D	E	B	B-II
Boost	MVA	rAd	rAd	MVA	rAd	rAd	rAd
Genes	B602L	B602L	B602L	B602L		B602L	**B602L**
	B646L		B646L		B646L		
	CP204L		CP204L		CP204L		
	E183L	E183L	E183L	E183L	E183L	E183L	**E183L**
	E199L	E199L			E199L	E199L	E199L
	EP153R	EP153R	EP153R	EP153R		EP153R	**EP153R**
	F317L	F317L			F317L	F317L	F317L
	*MGF505-5R*	MGF505-5R			MGF505-5R	MGF505-5R	MGF505-5R

^*a*^ In experiment 1, groups of five pigs were immunized with antigen pools A, B, C, D, and E, boosted 4 weeks later (day 28), and challenged with ASFV 6 weeks after the boost (day 73). In experiment 2, groups of five pigs were immunized with antigen pool B either tailored for genotype I or genotype II ASFV (pool B-I and B-II, respectively) and boosted 4 and 6 weeks later (days 28 and 41 post-prime). The individual ASFV genes in each pool and the principal type of vector used in boost are indicated. Italics highlight that MGF505-5R in pool A was vectored by rAd for both the prime and the boost. B602L, E183L, and EP153R are highlighted in bold in pool B-II to indicate these were genotype II open reading frames.

**Fig 1:**
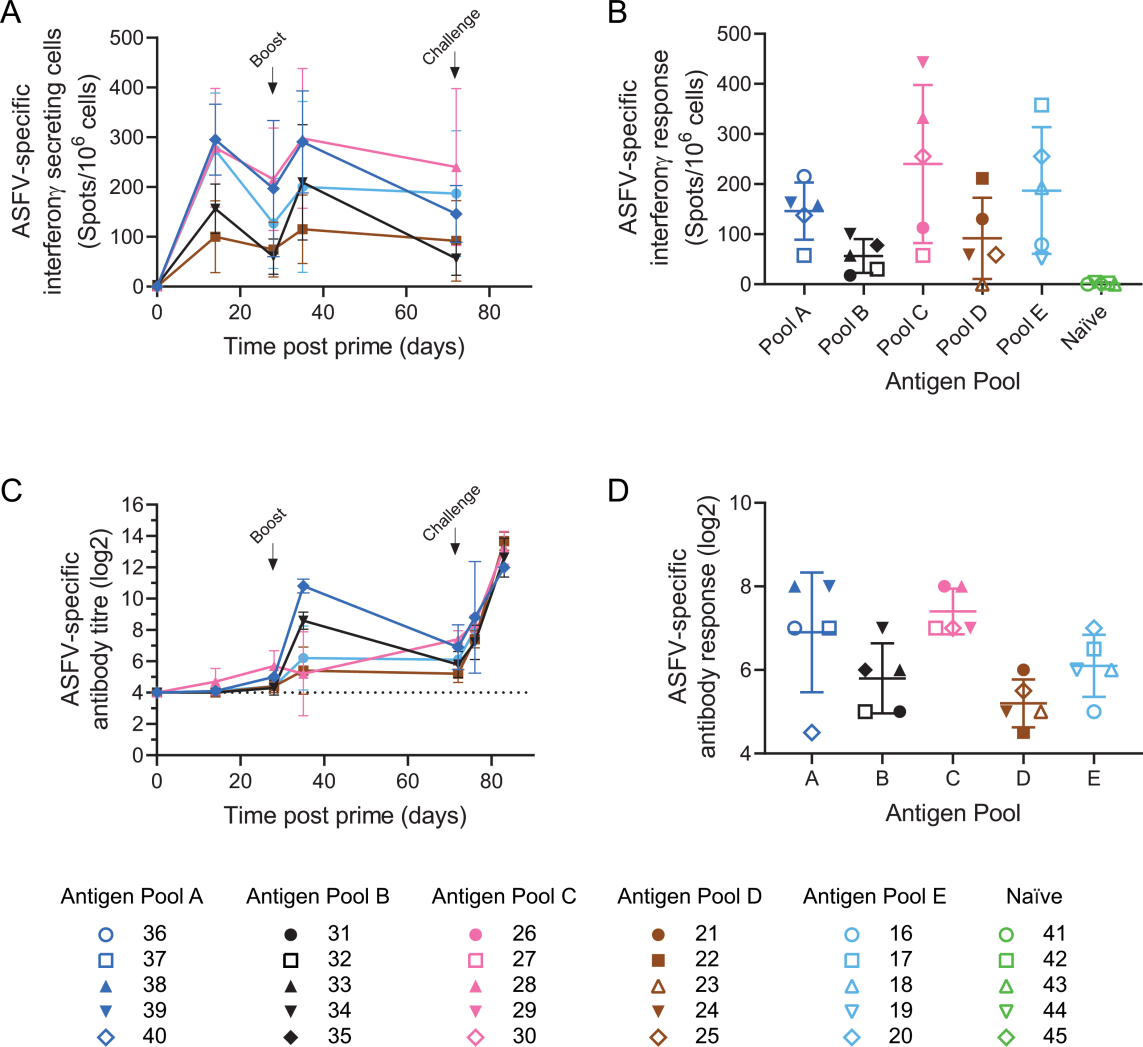
ASFV-specific immune responses in experiment 1. Groups of five pigs were immunized with antigen pools A, B, C, D, and E, boosted 4 weeks later (day 28) and challenged with ASFV 6 weeks after the boost (day 73). Blood samples were collected from the pigs on days 0, 14, 28, 35, and 71 after the first immunization. ASFV-specific cellular response induced by the antigen pools was determined by interferon-γ ELISpot after incubating PBMCs with whole virus (**A,B**). ASFV-specific antibody titers were determined by immunoperoxidase assay on Ba71v infected Vero cells (**C, D**). Data in panels A and C indicate the mean of each group, and error bars indicate the standard deviation from that mean. Panels B and D show immune responses in individual animals in each group 1 day before challenge (day 72), with mean and standard deviation from that mean. Solid symbols show animals that recovered after challenge and survived to the end of the experiment; open symbols indicate animals that reached their humane endpoint and were euthanized. Asterisks in panel A indicate significant differences between pool A and pools B and D at day 14 (repeated measures 2-way ANOVA; *P < 0.05), and in panel C, significant differences at day 35 between group A and groups B, C, D, and E (repeated measures 2-way ANOVA; *P ≤ 0.0404).

Immune responses were measured to whole virus and a selection of the individual antigens at different time points after immunization until the day before challenge. Cellular immune responses were measured using IFNγ ELISpot with whole virus as recall antigen (Fig. 1A), and antibody responses were assessed using a fixed cell ELISA (IPA) on ASFV-infected cells (Fig. 1C). The immune responses just before challenge for the individual animals are also shown in more detail (Fig. 1B and D). Cellular responses to a positive control stimulation with PHA were observed for all animals, albeit with variable levels at the different time points (Fig. S1A). ASFV-specific cellular immune responses were detected in all but one of the immunized animals (in pool D, Pig 23) 2 weeks after prime (day 14) and then throughout the course of the experiment. Taking each group as a whole, the only differences in the ASFV-specific cellular immune responses between the five antigen pools were 2 weeks post-prime where antigen pool A had higher responses than pool B (*P* = 0.0487) and pool D (*P* = 0.0163). There were no statistically significant differences between groups at any other time including the day before challenge (Fig. 1A and B). The number of ASFV-specific IFNγ-secreting cells 1 week post-boost (day 35) and pre-challenge (day 72) were not significantly higher than those observed prior to the boost for any of the antigen pools. In contrast, ASFV-specific antibody responses (Fig. 1C) were only detected 2 weeks post-prime in a few animals immunized with antigen pool A or antigen pool C, with all animals in these groups having serum ASFV-specific antibodies prior to the boost (day 28). Titers of ASFV-specific antibodies increased in groups A and B after the boost (day 35), but the results were more variable in the other groups. Antigen pool A led to higher ASFV-specific antibody titers than the other pools 1 week after boost (*P* ≤ 0.0404); however, these differences had disappeared by the time of the challenge (day 72). All animals had some ASFV-specific antibodies prior to challenge, but in some pigs, the titers were only just above background (Fig. 1D).

**Fig 2:**
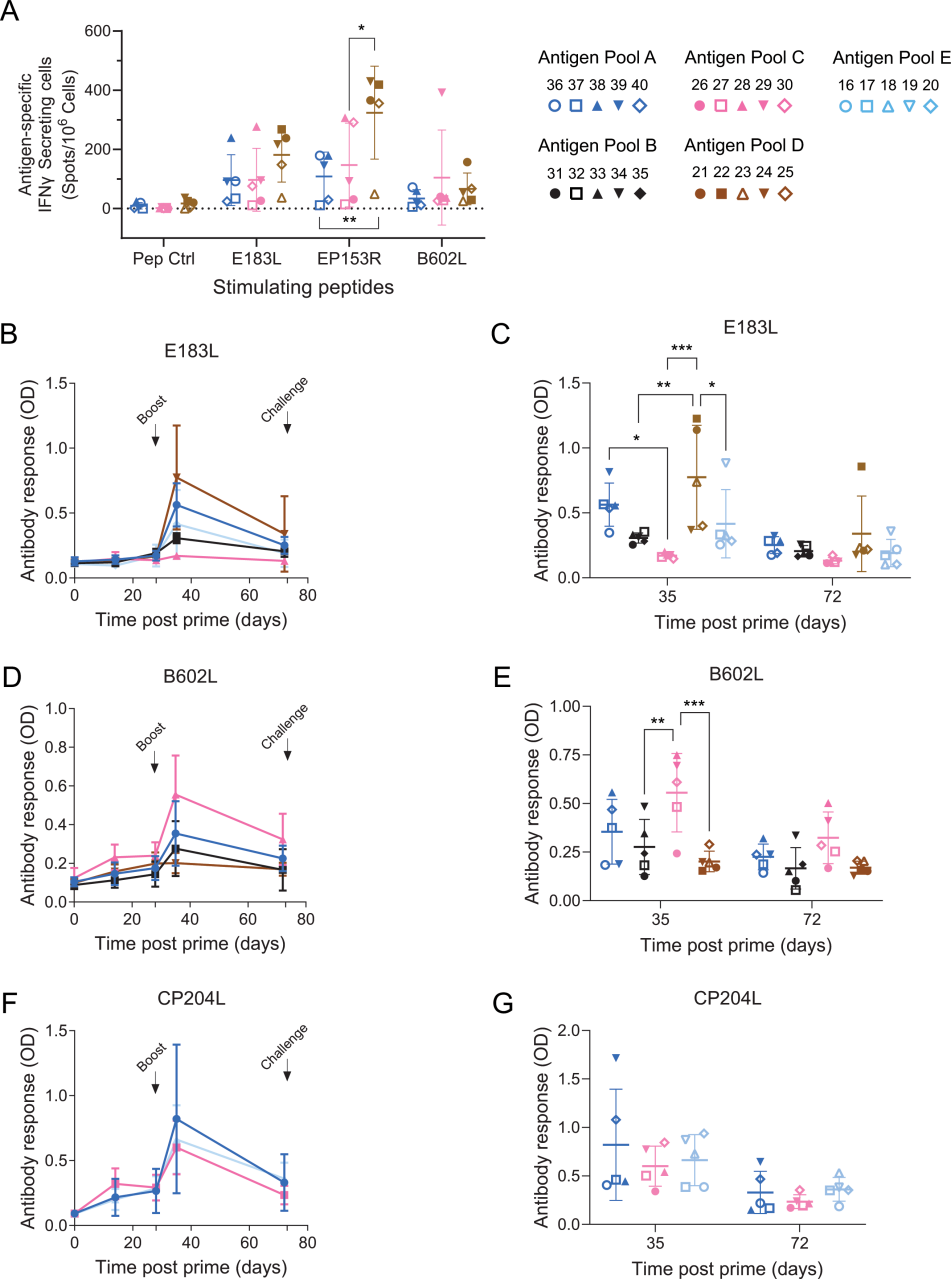
Antigen-specific immune responses in experiment 1. (A). IFNγ-producing cells at pre-challenge (day 71) after recall stimulation with peptides corresponding to the indicated antigens or a control (Pep ctrl). Responses from individual animals immunized with antigen pools A, C, and D are shown. Cells purified from blood collected on day 71 after immunization were incubated with the pools of peptides and numbers of IFNγ-producing cells determined by ELISpot. (B-G). Antigen-specific antibody titers were determined by indirect ELISA for E183L, B602L, and CP204L at days 0, 14, 28, 35, and 71 after the first immunization (OD, optical density). Data on the left panels represent the average and standard deviation (sd) at each time point for the different immunization pools; right panels show in more detail the individual values for each pig at post-boost (day 35) and pre-challenge (day 71), and bars indicate the mean and sd of the group. Individual animals are labeled with the same colors and shapes as in Fig. 1. Solid symbols show animals that recovered after challenge and survived to the end of the experiment; open symbols indicate animals that reached their humane endpoint and were euthanized. Asterisks indicate significant differences between two groups of animals (panel A, 2-way ANOVA, panels B-G repeated measures 2-way ANOVA; *P < 0.05, **P ≤ 0.01, ***P ≤ 0.001).

Antigen-specific cellular responses were tested in IFNγ ELISpot for the three antigens pB602L, pEP153R, and pE183L. Pools of peptides corresponding to these antigens or a control antigen (pA240R) were used in recall stimulation of pre-challenge PBMCs (day 72). Responses in groups immunized with different combinations with other antigens were tested, antigen pools A, C, and D (Fig. 2A). In comparison to the peptide control, stimulation with pE183L and pEP153R peptides induced an increase in IFNγ-producing cells in the majority of the animals in the three groups, with two of the five animals in pools A and C and one in pool D (Pig 23) not showing responses or very poor ones. The response to pEP153R was significantly higher in pool D that had only the three antigens as immunogens, and the magnitude of responses to pE183L also tended to be higher in pool D when looking at individual animals, than in pools A and C. Pools A and C had additional five and two antigens, respectively, which could indicate that the extra antigens in the immunization pools reduced the individual responses to pEP153R and pE183L. Pool C immunization was also different from pools A and D since it was via rAd homologous prime-boost, whereas pools A and D were heterologous immunizations with rAd/MVA. No significant differences were observed between pools A and C, suggesting that the heterologous immunization regimen did not improve cellular responses to the antigens, although pool A had three more antigens than pool C. Recall responses to pB602L in the different pools were very poor for the majority of the animals with the exception of one animal immunized with pool C and one animal immunized with pool D.

Antibody-specific responses were also quantified in indirect ELISA for antigens pE183L, pB602L, and pCP204L (Fig. 2B through G). pE183L was present in all immunization pools, and antibodies for this viral protein were detected with varying levels post-boost (day 35) in animals immunized with pools A, B, D, and E; however, pE183L antibody levels in animals immunized with pool B showed very low levels, and a specific antibody response was not detected in animals immunized with pool C (Fig. 2B and C). Interestingly, antibody responses to pE183L appeared to be higher in animals immunized with the heterologous prime-boost regime (pools A and D) when compared with those immunized with homologous rAd prime-boost (pools B, C, and E). When these groups were analyzed together, this difference was significant (unpaired *t*-test, *P* = 0.0002); however, no such difference was observed when pB602L levels were similarly compared between animals in pools A and D and those in pools B and C (*P* = 0.1580). This suggests that rAd-MVA prime and boost may perform better than rAd-rAd prime and boost for pE183L only, but this did not lead to significant differences in the overall ASFV-specific antibody response (Fig. 1C). Clearer differences were observed at post-boost (day 35), with significant differences for pE183L between groups and pool D showing a tendency for the highest response (Fig. 2C). However, pE183L-specific antibodies declined by the time before challenge day 72 to levels just above background except for one animal in pool D (Fig. 2C). pB602L antibodies were induced by all pools especially at post-boost but were very poor throughout immunization with pool D (Fig. 2D). At post-boost (day 35), there were significant differences between groups, with pool C inducing a higher antibody response than pools B and D (Fig. 2E), but no statistically significant differences resulted with pool A. In general, antibodies for pB602L also tended to decline between day 35 and day 72 in all groups. Antibodies for pCP204L, a highly immunogenic viral protein present in pools A, C, and E, were detected in all groups, with a tendency for higher levels at post-boost but also decreasing by pre-challenge day 72 (Fig. 2F and G), similarly to what was observed for the other specific antibodies. There were no significant differences between groups, indicating that the pool composition, or a heterologous (pool A) or homologous (pools C and E) immunization regimen does not seem to have an effect on the antibody responses for pCP204L.

### Protective efficacy of different pools of rAd against virulent genotype I ASFV

**Fig 3:**
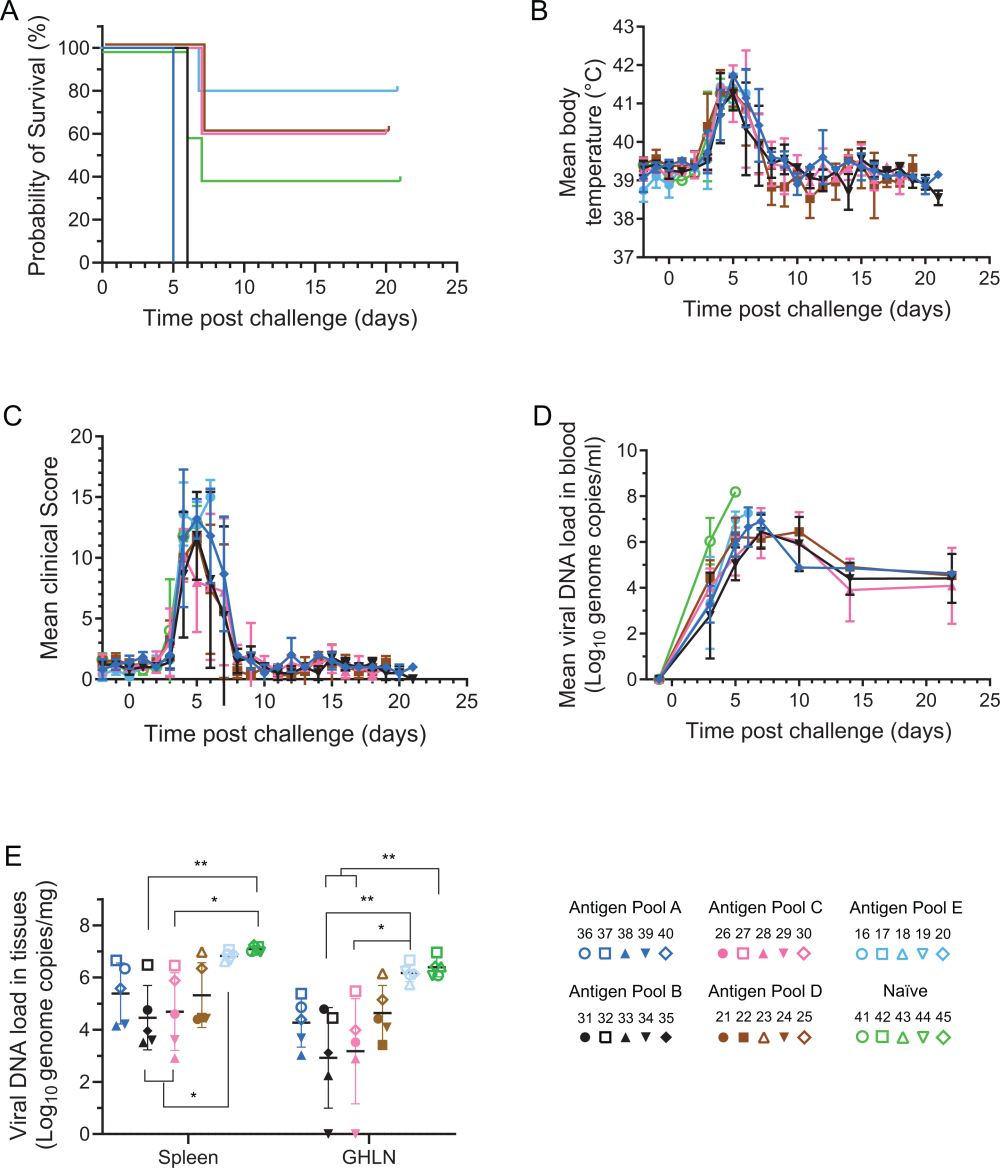
Experiment 1 clinical and virological data. Groups of pigs immunized with antigen pools A, B, C, D, and E, or naïve animals were challenged with OUR T88/1 on day 0. The percentages survival of each group are shown in panel A. Temperatures (**B**) and clinical signs (**C**) were recorded daily, and blood samples from virus detection were collected on the indicated days. Viral load was detected by qPCR alongside a reference and converted to the number of genome copies/mL of blood (**D**) or genome copies per mg of tissue (**E**) in spleen and gastro-hepatic lymph node (GHLN). Data indicate the mean of each group, and error bars the standard deviation from that mean. In panel E, solid symbols show animals that recovered after challenge and survived to the end of the experiment; open symbols indicate animals that reached their humane endpoint and were euthanized. Asterisks indicate a significant difference between two groups of animals (one-way ANOVA with Tukey’s post-test; *P < 0.05, **P ≤ 0.01).

Six weeks after boost, all of the animals, as well as a naïve group of five age-matched pigs from the same herd, were challenged in the rump with 10^4^ HAD_50_ OUR T1988/1. All animals began developing increased temperatures and clinical signs such as inappetence and lethargy between 3 and 4 days after infection with ASFV (Fig. 3B and C). Disease progression in the naïve animals was similar to that seen in previous experiments, and all animals were euthanized after reaching their humane endpoint 5 days post-challenge (Fig. 3A). All of the pigs immunized with pools of vectors showed at least 1 day of temperature above 41°C, and in a number of animals, this lasted several days. High temperatures in animals were coincident with a lack of interest in food and with lethargy. In order to mitigate clinical signs, a single treatment with a nonsteroidal anti-inflammatory and antipyretic drug flunixin meglumine (Finadyne, 2 mL per 45 kg bodyweight) was administered on day 5 post-challenge to all immunized pigs. Temperatures and clinical signs in animals immunized with antigen pool E remained high, and all of the animals reached their humane endpoints 6 days post-challenge (Fig. 3A). Results in the other groups were more variable, with animals either reaching humane endpoints 6 or 7 days post-infection or recovering and then remaining clinically normal until the end of the experiment. Viraemia levels were lower in all the immunized groups than in the naïve group at 5 days post-challenge, including with pool E that was not protective, and it persisted in the surviving animals albeit with lower levels until the end of the experiment (Fig. 3D). Four of five pigs immunized with antigen pool B, three out of five with antigen pools C and D, and only two out of five immunized with antigen pool A recovered (Fig. 3A). Only minor differences in temperature and clinical scores between the immunized pigs and the controls were observed during the acute phase of disease (Fig. 3B and C). A direct comparison of the survival curves for animals immunized with pool A in this experiment and our previous experiment where 100% recovered (24) showed the difference was statistically significant (Log-rank (Mantel-Cox), *P* = 0.0316). The reason for this difference is unknown, but possible explanations include a difference in the genetic background of the pigs (Landrace × Large white × Hampshire in this study compared with Landrace × Large white) and the difference in timing between boost and challenge (6 weeks in this experiment compared with 4 in the previous one). Comparison of the ASFV-specific immune response between the animals immunized with antigen pool A in the present study and our previous study revealed no difference in the number of IFNγ secreting cells (*P* = 0.1186, Welch’s t test) but reduced antibody titer in this experiment (*P* = 0.0023, Welch’s t test). However, neither of these are particularly satisfactory explanation as four out of five animals immunized with antigen pool B, which included six of the eight antigens in pool A, survived the challenge.

Postmortems were carried out on all the animals. Those that reached their humane endpoints had macroscopic lesions consistent with acute ASF, including hyperplasic and hemorrhagic lymphoadenopathy, particularly in the renal and gastro-hepatic lymph nodes (Fig. S2A). Animals also exhibited hyperemic splenomegaly and pulmonary congestion. Immunized pigs that recovered were clinically normal at the end of the study; however, a few animals exhibited mild hyperemic splenomegaly and showed lymph node hyperplasia and recovery from hemorrhagic lymphoadenopathy. Viral DNA load was determined in selected tissues, spleen, and gastro-hepatic lymph nodes (GHLN) (Fig. 3E). All immunized groups except with pool E showed a clear tendency for lower viral DNA loads in comparison to the naïve animals, with surviving animals in each group showing the lowest levels (Fig. 3E). Virus DNA levels in both spleen and GHLN were significantly lower for the group immunized with pools B and C in comparison to the naïve group and pool E.

### B602L, E183L, and EP153R drive a partially protective immune response against genotype I ASFV

Taken together with our previous data, the results from experiment 1 suggested that a combination of *B602L*, *E183L,* and *EP153R* plays an important role in the protection from fatal disease. These three antigens were present in all of the pools in which animals recovered in experiment 1. However, B602L and EP153R were used in combination with *EP364R*, *I329L*, *MGF360-11L*, *MGF505-4R,* and *MGF505-5R* in a previous study (24), and protection was not observed. Inclusion of E183L but absence of B602L and EP153R in pool E in this study also failed to confer protection. We have only seen protection in groups of animals that were immunized with all three antigens in combination. Additionally, animals immunized with a homologous rAd-prime/boost regime recovered after a challenge showing that heterologous rAd-prime MVA-boost was not required for protection, despite the apparent differences in anti-pE183L antibody levels. ASFV-specific (Fig. 1B and D) and antigen-specific immune responses (Fig. 2C, E and G) measured prior to challenge in protected pigs are shown as solid symbols, and those in pigs that were not protected, as open symbols. There were no differences in the measured immune responses in the group of pigs immunized with antigen pool E that were not protected and the other groups where variable protection was observed. Interestingly, the animals immunized with pool D that recovered appeared to have higher cellular responses to pE183L and pEP153R (Fig. 2A); however, other than this, there was no clear correlation between the magnitude of virus- or antigen-specific responses to protection, when looking at the responses in individual animals that were protected and not protected. Taken together, this suggested that the magnitude of the measured immune responses did not correlate well with the clinical outcomes of the pigs either individually or as groups. Additional studies looking more in depth at the immune responses after vaccination are necessary.

### Comparative immune responses induced by genotype I and genotype II tailored pools of rAd

The next experiment was designed to test the ability of one of the pools of antigens to protect pigs against the virulent genotype II strain circulating across Europe and Asia. Antigen pool B was selected for this experiment as the animals immunized with this pool exhibited the lowest viral DNA loads in blood 3 and 5 days post-infection, comprised a homologous prime-boost regime and had the greatest number of survivors. The three antigens present in all of the protected groups, pB602L, pE183L, and pEP153R, were the most genetically divergent of the six antigens (Table 2). Therefore, new replication-deficient adenoviruses expressing the genotype II Georgia 2007 *B602L*, *E183L,* and *EP153R* genes were generated (Fig. S3 and S4) and used in experiment 2. These were combined with adenoviruses expressing genotype I *E199L*, *F317L,* and *MGF505-5R* to create antigen pool B-II.

**TABLE 2:** Identity between protein sequences of genotype I and genotype II antigens.

Antigen^*b*^	Identity (%)	Similarity (%)
pB602L	78.0^*a*^	78.3
pE183L	96.2	96.2
pE199L	98	98.5
pEP153R	52.4	66.3
pF317L	99.7	99.7
pMGF505-5R	98.4	98.8

^*a*^ Differences due to 141 and 4 aa insertions, as well as three other differences in central variable region. Remainder of the protein is identical.

^*b*^ All genotype II protein sequences were derived from georgia 2007/1 and genotype I sequences were from OUR T1988/3, except for pEP153R, which was from Benin 1997/1.

**Fig 4:**
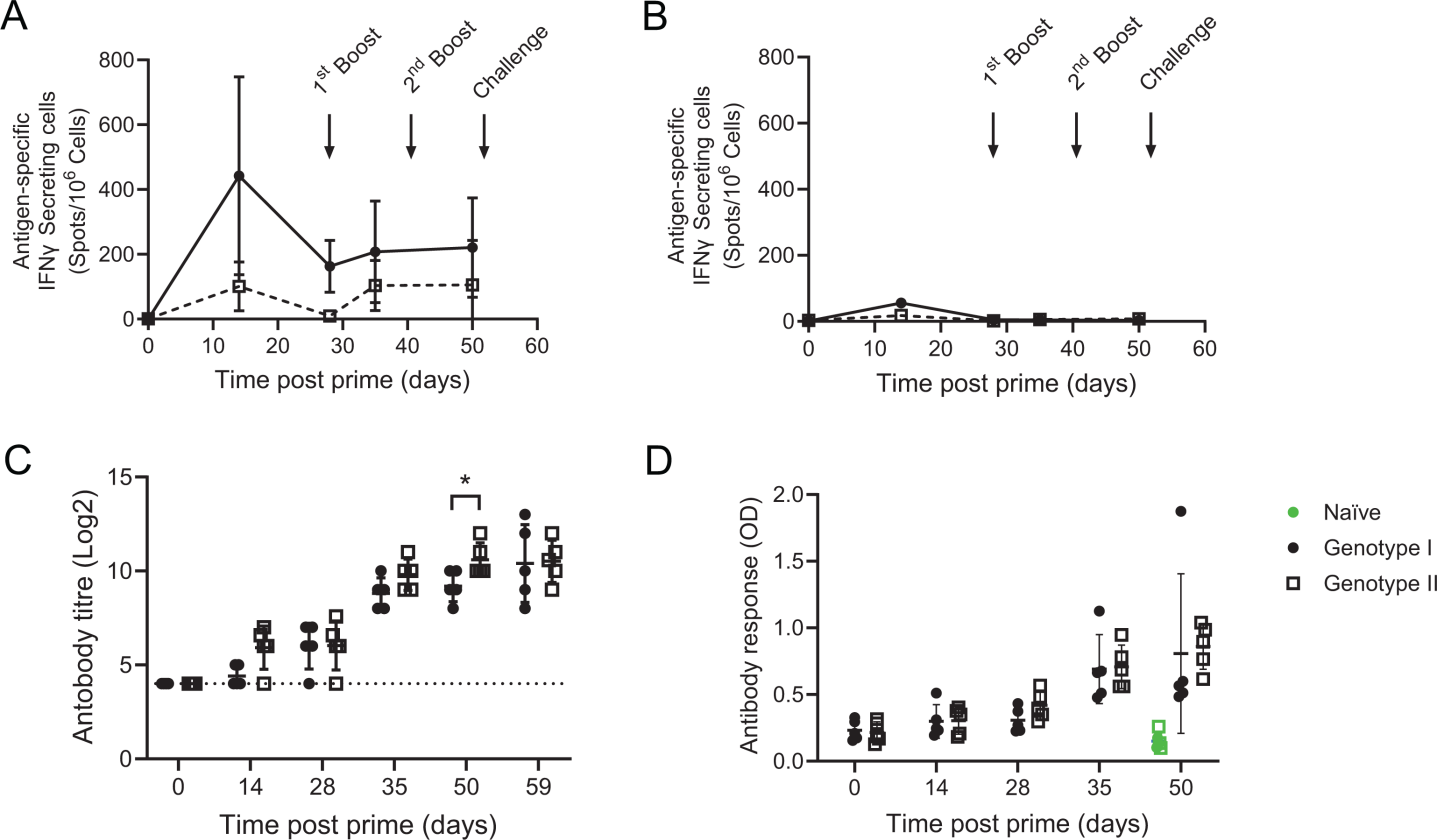
ASFV-specific immune responses in experiment 2. Groups of five pigs were immunized with antigen pool B either tailored for genotype I or genotype II ASFV (solid lines and dashed lines, respectively) and boosted 4 and 6 weeks later (days 28 and 41 post-prime). Blood samples were collected from the pigs on the indicated days. Cells purified from blood were incubated with genotype I (panel A) or genotype II (panel B) virus, and ASFV-specific IFNγ cells were determined by ELISpot. ASFV-specific antibody titers were determined by immunoperoxidase assay on Ba71V-infected Vero cells (**C**), and B602L-specific antibody responses were determined by indirect ELISA (**D**). Data show the individual values for each pig in the groups, and bars indicate the mean and standard deviation of that group. Dotted lines on panel C indicate the limits of detection of the immunoperoxidase assay.

Cellular immune responses to whole virus were tested by IFNγ ELISpot at different times after immunization and until 2 days before challenge (days 0, 14, 28, 35, and 50). There was a clear induction of IFNγ-secreting cells that responded to stimulation with genotype I virus in both immunization groups, but the response from the animals immunized with the genotype I tailored vaccine was clearly higher than those immunized with the genotype II vaccine (Fig. 4A). Furthermore, the responses to genotype II virus were very poor or non-existent throughout the whole immunization period (Fig. 4B). At all time points, clear responses to a positive control stimulation with PHA were observed (Fig. S1 B), attesting the good condition of the cells.

Although analysis of serum taken 7 days post-boost (day 35) showed the presence of ASF- and pB602L-specific antibody responses (Fig. 4C and D, respectively), due to the lack of a clear cellular response to stimulation with live Georgia 2007/1 (Fig. 4B), the animals were immunized 41 days after the initial prime and 13 days after the first boost with a further boost. All animals were immunized with a pool of adenoviruses expressing either genotype I or genotype II genes for the three key antigens identified in the previous experiment, *B602L*, *E183L,* and *EP153R*. The extra boost did not lead to an increase in ASFV-specific T-cell responses, although, interestingly, the antibody response in the animals immunized with genotype II vaccine was slightly higher before challenge than that of the genotype I immunized animals (Fig. 4C). The antibody response was also tested on MA104 clone 1 cells infected with genotype II Georgia 2007/1 (Fig. S5), and although the sensitivity was reduced compared with Vero cells infected with Ba71v at earlier time points after immunization (days 14 and 28), the data showed that the genotype I and II tailored vaccines induced antibody responses capable of cross-reacting with cells infected with genotype I and genotype II ASFV.

**Fig 5:**
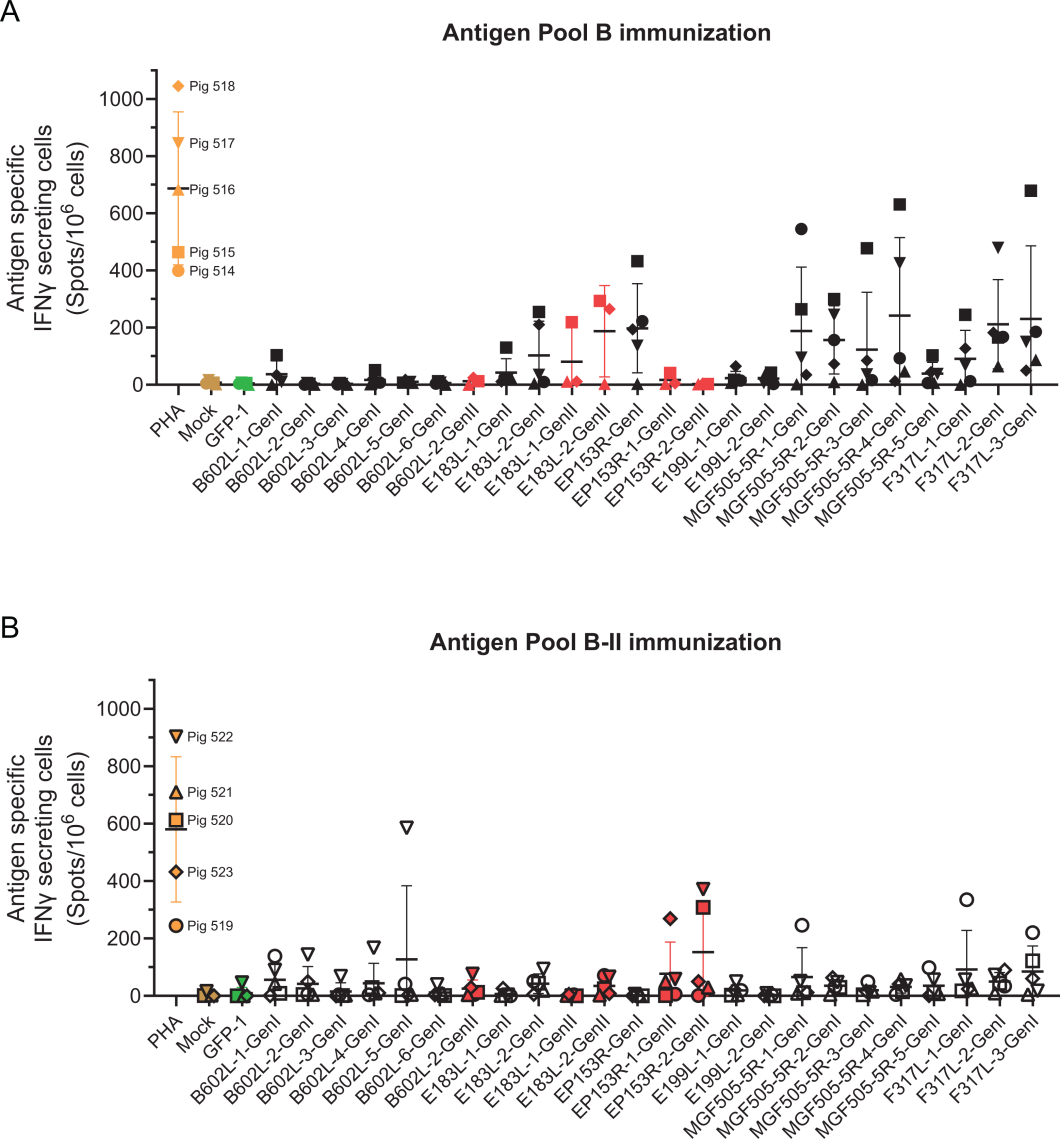
Antigen-specific cellular immune responses in experiment 2. Groups of five pigs were immunized with antigen pool B either tailored for genotype I (panel A) or genotype II ASFV (panel B). Cells collected 2 days pre-challenge (50 days post-prime) were incubated with pools of peptides corresponding to the genotype I (black symbols) or genotype II (red symbols) antigens or GFP (green symbols) as a negative control, and PHA as a positive control (brown symbols). Depending on the size of the proteins, the peptides were divided into smaller pools with a maximum of 27 peptides in each. Numbers of IFNγ-producing cells were determined by ELISpot. Each animal was assigned a different symbol, which is indicated to the right of the PHA results, responses from genotype I immunized animals are shown with filled symbols and responses from genotype II animals with open ones.

Antigen-specific cellular immune responses were tested pre-challenge using IFNγ ELISpot (Fig. 5). For proteins with significant differences between genotypes, pools of peptides tailored to genotype I or genotype II were also used in stimulation for selected animals (Fig. S6). In animals immunized with both antigen pool B and pool B-II, the responses to pB602L and pE199L peptides were, in general, poor with the exception of one animal (Pig 522), which responded to several of the B602L peptide pools (Fig. 5B). Peptides corresponding to the C-terminal half of pE183L triggered responses in a couple of the animals (Pigs 515 and 518), with both genotype I and II tailored peptides giving similar results (Fig. 5A). Immune responses to pEP153R were detected in both groups of animals; however, unlike pB602L and pE183L, the immune response was genotype-specific. Pigs immunized with genotype I *EP153R* did not cross-react to peptides from the genotype II protein (Fig. 5A) and vice versa (Fig. 5B), consistent with the low amino acid identity between the two orthologs. Responses were also observed against pMGF505-5R and pF317L peptide pools, albeit varying between animals and peptide pools. Looking at the responses from individual animals, Pig 516’s responses were very poor to all the peptides (a similar result was observed in ELISpot after virus stimulation, Fig. 4), which was not due to a poor condition of the cells since PHA stimulation clearly led to many IFN-producing cells (Fig. 5A). Pig 515, on the other hand, was the animal that responded most frequently to the peptide pools and with the highest magnitude in most stimulations (Fig. 5A). Hence, all antigens in antigen pool B immunization effectively primed T-cell responses, although for pE199L, these were especially poor. Regarding pool B-II immunized animals, antigen-specific responses were poor except for pigs 519 and 522, which were the best responders in general (Fig. 5B). Pig 519 showed increased numbers of IFNγ-producing cells in response to pB602L, pMGF505-5R, and pF317L peptide and Pig 522 in response to pB602L and genotype II pEP153R. Pigs 523 and 520 also had clear responses to peptides corresponding to pEP153R of genotype II, and pig 520 also responded to pF317L peptides. Thus, in pigs immunized with pool B-II, pB602L, pEP153R, and pF317L were the main antigens inducing IFNγ-producing cells. It is interesting that antigen-specific responses were detected in pool B-II immunized animals but not in live genotype II virus (see Fig. 4B). Taken together, this showed that the vectors induced antigen-specific cellular responses in the pigs, but this did not lead to strong responses to genotype II virus.

### A genotype II tailored pool of rAds does not protect against genotype II challenge

**Fig 6:**
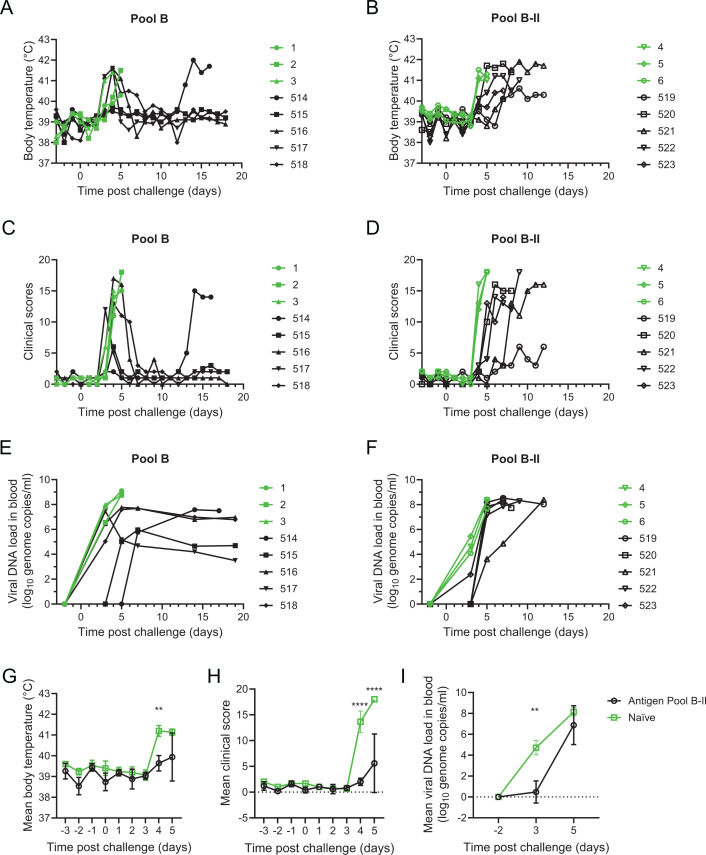
Experiment 2 clinical and virological data. Groups of pigs immunized with antigen pool B (solid symbols) or antigen pool B-II (open symbols) or naïve animals (green symbols) were challenged with OUR T88/1 (solid symbols) or Georgia 2007/1 (open symbols) on day 0. Temperatures (**A, B, G**) and clinical signs (**C, D, H**) were recorded daily, and viral DNA in blood was determined by qPCR on the indicated days (**E, F, I**). Panels G, H, and I show the mean of the two groups of pigs infected with Georgia 2007/1 up to 5 days after infection, and error bars the standard deviation from that mean. Statistical differences between the naïve and immunized groups were determined by RM 2-way ANOVA in panels G, H, and I (**P < 0.01, ****P < 0.0001).

Fifty-two days after the first immunizations, the group of animals immunized with the genotype I antigens along with a group of three age-matched naïve controls were then challenged with the virulent genotype I isolate ASFV OUR T1988/1. Likewise, the animals immunized with the genotype II antigens as well as a control group were challenged with the virulent genotype II isolate Georgia 2007/1. Back titration confirmed a dose of 10^4^ HAD_50_ of OUR T1988/1 per pig and a dose of 10^2.6^ HAD_50_ of Georgia 2007/1 per pig. Clinical signs began to appear in the animals immunized with the genotype I vaccine 3 to 4 days post-challenge (Fig. 6A and C), whereas in the pigs immunized with genotype II vaccine, clear clinical signs did not appear until 5 days after infection (Fig. 6B, D, G, and H). Both groups of naïve pigs displayed clinical signs typical of acute ASF 4 days post-infection with one pig infected with OUR T1988/1 reaching its humane endpoint on this day. The remaining control pigs showed high fever, inappetence, and lethargy 5 days post-infection and were therefore euthanized. Clinical signs 4 and 5 days post-infection were significantly different between the naïve and immunized groups of pigs challenged with Georgia 2007/1 (Fig. 6G and H), but not the two groups of pigs challenged with OUR T1988/1. Four of the five vaccinated animals that were challenged with OUR T1988/1 suffered a transient fever lasting 2 to 3 days but then began to recover and were clinically normal 7 to 8 days post-infection (Fig. 6A and C). Pig 514 in this group had shown few clinical signs initially but developed a temperature of 40.8°C 13 days post-infection and showed clinical signs consistent with acute ASF. This animal was euthanized 16 days post-infection with OUR T1988/1; however, the remainder of the animals in this group were clinically normal when the experiment ended 2 days later. This delay in the appearance of clinical signs had not been seen in previous experiments, and there were no obvious differences in the measured immune responses for Pig 514 when compared with the other pigs in the group that may have explained this difference. Note that none of the animals were treated with anti-inflammatory drugs showing that this was not essential for the animals to avoid reaching the humane endpoints of the study. In the group of animals immunized with the genotype II antigens, clinical signs consistent with acute ASF first appeared between 5 and 8 days post-infection with one pig showing limited clinical signs. All animals were euthanized before the end of the experiment. This showed that the genotype II vaccination was able to delay the appearance of clinical signs but was not capable of preventing acute ASF.

The appearance of virus in the blood is generally associated with the appearance of clinical signs (Fig. 6E and F), and consistent with this, only one animal immunized with the genotype II antigens had detectable virus in the bloodstream 3 days post-challenge, whereas all of the controls were PCR-positive (Fig. 6F and I). Five days after the challenge, there was no difference in the viral load between the vaccinated and control pigs. All of the animals that survived the genotype I challenge had virus circulating in the blood when the experiment ended (Fig. 6E).

Postmortem analysis of the naïve pigs infected with both OUR T1988/1 and Georgia 2007/1 revealed typical lesions and changes in lymphoid tissue, mild-to-moderate hydropericardium, and pulmonary congestion and hemorrhages (Fig. S2B). Animals immunized with antigen pool B that were challenged with OUR T1988/1 showed relatively mild lymphoadenopathy and splenomegaly with the exception of pig 514 that was culled 16 days after challenge, which had lymphoadenopathy and petechiae across a broader range of lymph nodes. The pigs immunized with antigen pool B-II and that were challenged with Georgia 2007/1 showed more significant external and internal lesions with frequent bloody conjunctiva, skin cyanosis, and hemorrhages as well as more extensive lymphoadenopathy. This was consistent with the longer course of disease and prolonged period of clinical signs. Pig 519’s behavior was relatively normal but showed hemorrhagic lymphoadenopathy in multiple lymph nodes as well as extensive subcutaneous hemorrhages consistent with subacute ASF. Viral load was determined in selected tissues, and in both spleen and gastrohepatic lymph node, a significantly lower viral load was present in the animals immunized and challenged with genotype I that were culled at the end of the experiment in comparison to the naïve group that were culled 4 to 5 days post-challenge (Fig. S7). Pig 514 that developed disease had correspondingly the highest viral loads of the group in both tissues and Pig 519 that had the lowest clinical score of the group immunized with pool B-II had the lowest viral load in the spleen but not in the GHLN. There were however no significant differences in the viral loads in tissues of the animals immunized with pool B-II and the corresponding naïve control group.

## DISCUSSION

Previous experiments in our laboratory had demonstrated that a pool of virally vectored ASFV genes could protect pigs against severe disease caused by genotype I virus. Improving vaccine performance requires a better understanding of the protective immune response, as well as rationalizing the combination of antigens and vectors. To this end, five different combinations of the original eight antigens (B602L, B646L, CP204L, EP153R, E199L, E183L, F317L, and MGF505-5R) were tested, and all but one (lacking B602L and EP153R) resulted in some protection against challenge with the highly virulent genotype I isolate OUR T1988/1. Protection was also observed in groups of pigs primed and boosted with adenoviruses alone, suggesting that homologous prime boost was sufficient, and heterologous boost was not required.

The present study did not replicate the 100% protection after immunization with the pool of eight antigens as seen previously (24). Contributing factors to these differences could be the different genetic backgrounds of the animals and the different intervals between prime, boost, and challenge. We also used humane endpoints in all of our studies rather than allowing pigs to become moribund or die, and as all of the pigs developed clinical signs after challenge, this introduces an additional variable. It is also important to note that we observed 33% protection with a lower dose of the viral vectors in our previous study (24), which is similar to the 40% protection observed in this study. In our previous publication, we concluded that 100% protection was related to the higher dose of vectors used in the second experiment (24); however, in light of the 40% protection observed here, this conclusion may not be sustainable. We do not believe the virus genes were unstable in the vectors since expression in transduced cells had been confirmed by us previously (24, 30), and the vectors induced comparable immune responses in all of the experiments. Co-infections were also unlikely since the animals were obtained from a high health herd, had been vaccinated against several pig pathogens, and the farm reported free from PRRSV. Ultimately, we were able to demonstrate reproducible protection of 80% of the animals with six of the antigens that produced 100% protection in our previous study.

It is likely that a combination of B602L, E183L, and EP153R is particularly important for protection for the following reasons. Three of five pigs immunized with antigen pool D that comprised B602L, E183L, and EP153R alone recovered after challenge. In our previous experiment, the pool of eight antigens gave 100% protection and a separate pool that lacked B602L, E183L, and EP153R was not effective at all (24). In addition, a separate pool of eight antigens including both B602L and EP153R did not protect (24), and in the present study, antigen pool E that lacked B602L and EP153R, but not E183L, was also not protective. Although we cannot rule out the possibility that some of the other antigens in these last two pools were inducing disease enhancement that confounded the protection conferred by E183L alone, or B602L and EP153R in combination, we judge this to be unlikely. *E183L* encodes for p54 a well-characterized structural protein that is a potential target for neutralizing antibodies (18, 31). E183L is also a component of both DNA and baculovirus vaccination regimes that induce partial protection against genotype I challenge (18, 19); therefore, it appears that *E183L* encodes for an important protective antigen against genotype I ASFV. *B602L* encodes for a late viral protein that localizes to the cytoplasm and acts as a chaperone for the major capsid protein during virus assembly (32, 33), whereas the *EP153R* gene encodes for a C-type lectin (34) that comprises part of the genetic loci corresponding to serological specificity (35). pEP153R is one the most variable ASFV proteins and has two regions containing T-cell epitopes (36), which are only 44% and 26% identical between the genotype I and genotype II sequences. The cellular immune response to genotype I virus, in the animals immunized with Pool B-II with the genotype II antigens, was reduced in comparison with the response of the animals immunized with pool B with genotype I antigens. This showed that one or more of pB602L, pE183L, and pEP153R were important antigens mediating the ASFV genotype I-specific T-cell response. This, coupled with the observation that animals immunized with the genotype II antigens did not recognize peptides derived from genotype I pEP153R, suggests this protein may play an important role in driving the genotype I ASFV-specific T-cell response. A more detailed analysis of the response to individual antigens may identify immunodominant regions within the proteins that drive the cellular immune response to OUR T1988/1 and would help further rationalize the composition of the vaccine.

Outbreaks of genotype I ASFV have been reported in western and central African countries with a combined population of more than 570 million people (37–39); however, genotype II ASFV is of major global concern due to its spread across Europe, Asia, and Oceania since 2007 and appearance in the Caribbean in 2022. Experiments with live-attenuated viruses have shown that results cannot necessarily be transferred between different ASFV genotypes (3, 11, 40). Consistent with this, our data showed that a pool of adenovirus vectored genotype I *F317L*, *MGF505-5R,* and *E199L* along with genotype II *B602L*, *E183L,* and *EP153R* led to the pigs developing acute ASF after challenge with Georgia 2007/1, despite a delay of 1 to 2 days in the appearance of clinical signs and viraemia. The genotype II tailored pool of antigens induced antigen and ASFV-specific antibody responses as well as a cellular immune response that recognized genotype I ASFV. However, no cellular immune response was detected in PBMCs stimulated with Georgia 2007/1 despite the inclusion of an additional boost with rAds expressing genotype II tailored *B602L*, *E183L,* and *EP153R*. The absence of a genotype II response could be due to a number of factors. It is unlikely that the Georgia 2007/1 strain is capable of blocking the secretion of IFNγ *per se* as the same preparation of virus was used in other experiments where IFNγ secretion was detected by both ELISpot and flow cytometry (41). In addition, Georgia-specific cellular immune responses induced by both plasmid DNA (25, 42) and MLVs (5) have been previously reported. A more detailed analysis of the immune cells that respond to the virus may yield additional insights, for example, CD4 + CD8α + T cells have recently been implicated as playing a central role in the ASFV-specific cellular immune response (43).

The delay in virus DNA detection in blood and clinical signs after Georgia 2007/1 challenge of the group of pigs immunized with the genotype II tailored pool is therefore likely linked to the antibody responses induced by the pool of six vectors. Neutralization assays of field isolates in macrophages are technically challenging (44), and we have been unable to demonstrate neutralizing or enhancing activity *in vitro*. Complement-dependent and antibody-dependent cellular cytotoxicity effector functions have been described (45, 46) but are poorly understood. The target(s) and effector functions of the ASFV-antibody response induced by the six antigens may represent important parts of the puzzle required for the development of an effective ASF subunit vaccine. Clarifying the mechanism(s) may enable the identification of the antigens that contributed to the delay in viremia and clinical signs after Georgia 2007/1 challenge, as well as the identification of further protective antigens.

Adenoviruses expressing *B602L*, *E183L*, and *EP153R*, as part of large fusions with other ASFV antigens, did not protect domestic pigs or wild boar against genotype II challenge (28, 29), although it is unclear if these immunization regimes induced ASFV-specific cellular immune responses. Two pools of adenovirus vectored antigens that included either B602L or E183L induced antigen-specific responses, but ultimately, it did not protect pigs after challenge with Georgia 2007/1 (27). Unfortunately, ASFV-specific cellular immune responses were not measured, and therefore, it is not possible to draw comparisons with our data to make inferences about whether individual antigens may contribute to the virus-specific response. Strikingly, pigs in a farm setting that were immunized with a HuAd2-vectored combination of *CP204L*, *B602L*, *B646L*, *E183L,* and *EP402R* delivered by combined intranasal and intramuscular inoculation survived an outbreak of ASF, where pigs immunized with a control antigen did not (47). The principal differences in the antigens between this and our study are the lack of *EP153R*, the addition of *EP402R* that encodes for the viral CD2v protein and is an important protective antigen in MLV models (48), and the inclusion of *CP204L* and *B646L* that we found was not necessary for protection against genotype I ASFV. Additionally, in our study, the animals were challenged via intramuscular inoculation, whereas in Liu et al.’s study, it was through contact with outbreak animals, and therefore, it is not possible to assess the level of exposure of the immunized animals to virus and draw a comparison with our study, especially since virus-specific antibody responses are not being shown. Recent work has also suggested a potential role for MGF505-7R, M448R (42), and A104R (49) in protection against genotype II ASFV, and therefore, it would be interesting to see if the addition of adenoviruses expressing these genes enhances the protection we observed against genotype II ASFV in our direct challenge model.

Developing vaccines to control the ASF panzootic has been further complicated by the detection in the field in China of a circulating virulent genotype I and II ASFV recombinant, which is able to evade protection conferred by immunization with a genotype II LAV (50). This highlights the need to identify which ASFV antigens may confer protection against different circulating ASFV strains to produce safe and effective subunit vaccines.

## MATERIALS AND METHODS

### Viruses and cells

Growth and titration of the Vero cell tissue cultured adapted Ba71V and virulent OUR T1988/1 ASFV isolates have been described previously (24, 30). Georgia 2007/1 isolate of ASFV (51) was cultured and titrated on porcine bone marrow-derived macrophages (24). MA104 clone 1 (ATCC CRL-2378.1) cells were cultured in MEM medium supplemented with 10% fetal bovine serum, 1 mM sodium pyruvate, 100 IU/mL penicillin, and 100 µg/mL streptomycin and infected with ASFV as described (52). Challenge doses for animal experiments were confirmed by back titration on porcine bone marrow-derived macrophages. Mock inoculums were prepared from uninfected bone marrow cultures from the same animals as those used to propagate virus stocks. Peripheral blood mononuclear cells (PBMC) were cultured in RPMI GlutaMAX (Thermo Fisher), 25 mM HEPES supplemented with 10% fetal calf serum, 1 mM sodium pyruvate, 50 µM 2-mercaptoethanol, 100 IU/mL penicillin, and 100 µg/mL streptomycin (RPMI/10). ASFV genomic DNA was detected using real-time quantitative polymerase chain reaction (qPCR) (24, 53).

### Recombinant vectors

Vectors expressing genotype I ASFV genes *B602L*, *B646L* (p72), *CP204L* (p30), *E183L*(p54), *E199L*, *EP153R*, *F317L,* and *MGF505-5R* have been described previously (24, 30). Genotype II ASFV open reading frames (ORFs) for *B602L*, *E183L,* and *EP153R* were derived from the Georgia 2007/1 strain of ASFV and were codon-optimized for expression in *Sus scrofa*, synthesized and then cloned into pcDNA3.1zeo(+) (Thermo Fisher). ASFV ORFs were then subcloned into transfer plasmids for making recombinant replication-deficient human adenovirus 5 (rAd) using standard techniques. Purified viral vectors were generated by the Pandemic Sciences Institute Viral Vector Core Facility (Oxford, UK).

### Animal experiments and ethics statement

All of the animal experiments were carried out under the Home Office Animals (Scientific Procedures) Act (1986) (ASPA) and were approved by the Animal Welfare and Ethical Review Board (AWERB) of The Pirbright Institute and the AWERB of the Animal and Plant Health Agency (APHA), Weybridge. Prime and boost with viral vectors were carried out at APHA. The animals were housed in accordance with the Code of Practice for the Housing and Care of Animals Bred, Supplied or Used for Scientific Purposes, and bedding and species-specific enrichment were provided throughout the study to ensure high standards of welfare. Through careful monitoring, pigs that reached the scientific or humane endpoints of the studies were euthanized by an overdose of anaesthetic. All procedures were conducted by Personal License holders who were trained and competent and under the auspices of Project License PPL70/8852.

Female Landrace × large white × Hampshire pigs were obtained from a high health farm in the UK and randomly assigned to each group prior to immunization. Piglets had been vaccinated against PCV2 (subunit) - (all piglets), and sows and gilts were vaccinated against *E. coli* (multiple subunits of adhesins and a toxin), Erysipelas (inactivated), and Parvovirus (inactivated). The farm has been PRRSV-free since at least 2019.

#### Experiment 1

Five groups of five, 8-week-old, female outbred pigs were inoculated intramuscularly in the neck with pools of between three and eight rAd expressing ASFV ORFs (pool A, pigs 36 to 40; pool B, pigs 31 to 35; pool C, pigs 26 to 30; pool D, pigs 21 to 25; and pool E, pigs 16 to 20) (Table 1). Each rAd expressing an ASFV gene was administered at a dose of 1.5 × 10^10^ IU; therefore, all animals were primed with a maximum of 4 × 10^11^ IU rAd per pig. Four weeks later, the pigs were inoculated in the same site with rAd or MVAs expressing the same ORFs (see Table 1). Each MVA expressing an ASFV gene was administered at a dose of 2 × 10^8^ pfu, and each rAd at a dose of 1.5 × 10^10^ IU. Five weeks after the boost, all of the pigs, as well as an age-matched naïve group of five animals (pigs 041 to 045), were challenged by the intramuscular route in the rump with 10,000 50% Hemadsorption dose (HAD_50_) of OUR T1988/1. Blood samples were collected before each immunization (days 0 and 28), after the prime and boost (days 14 and 35), before challenge (day 72) as well as after selected intervals after challenge with ASFV. Due to an unforeseeable delay in the availability of the isolation facilities to host the animals after challenge in this experiment, these were challenged at a later date than 50 days after prime as originally planned.

#### Experiment 2

Two groups of five, eight-week-old, female outbred pigs were inoculated intramuscularly in the neck with a pool of six rAd each expressing an ASFV ORF (pool B, pigs 514 to 518; pool B-II, pigs 519 to 523). Four weeks later, the pigs were boosted at the same site with the same pool of rAd. All of the animals were given a further boost 2 weeks after the first boost with a pool of three rAd each expressing either the genotype I (pool B) or genotype II (pool B-II) *B602L*, *E183L,* and *EP153R* genes. Each rAd was administered at a dose of 1.5 × 10^10^. Two weeks after the second boost, the group of pigs immunized with pool B, as well as three age-matched naïve pigs, were challenged by the intramuscular route in the rump with 10,000 HAD_50_ OUR T1988/1. The group of pigs immunized with pool B-II, as well as three age-matched naïve pigs, were challenged by the intramuscular route in the rump with 10,000 HAD_50_ Georgia 2007/1. Blood samples were collected before each immunization (days 0 and 28), after the prime and first boost (days 14 and 35). Blood was also collected before challenge (day 52) as well as after selected intervals after challenge with ASFV.

### Interferon-gamma ELISpot

PBMC were purified from heparinized blood using histopaque (Sigma -Aldrich, Gillingham, UK) gradients and then washed extensively with PBS. The response to ASFV was analyzed after *in vitro* stimulation of fresh cells; however, the response to peptides was analyzed using cells that had been frozen, where viability was ≥90% after thawing. Only samples from animals for which there was sufficient material to test antigen-specific responses at all relevant time points were analyzed in this way. PVDF membrane multiwell plates (Millipore, Abingdon, UK. MAIPS4510) were coated overnight at 4°C with 4 µg/mL anti-porcine IFNγ (P2F6) in 0.5 M carbonate-bicarbonate coating buffer and then washed with PBS. Cells were plated in duplicate at two different dilutions, typically 5 × 10^5^ and 2.5 × 10^5^ per well in RMPI/10. Cells were then incubated overnight in a final volume of 200 µL with media alone, 0.5% DMSO, 10^5^ HAD_50_ of OUR T1988/1 or an equivalent volume of mock inoculum, or 2.5 µg/mL phytohemagglutinin (PHA) or peptide pools. Twenty-mer peptides overlapping by 10 amino acids supplied at 1 to 3 mg scale (Mimotopes) were used in experiment 1. EP153R was covered by a total of 11 peptides and E183L by 18 peptides. These werea dded to the cells at a final concentration of 10–30 µg/mL of each peptide (1–3 µg/peptide added to the wells; 11–33 µg total peptide mass added for EP153R, and 18–54 µg for E183L). For B602L, there were 67 peptides in the pool, added to the cells at a final concentration of 5–15µg/mL (0.5–1.5 µg/peptide added to the wells, 33.5–100.5 µg total peptide mass). The molecular mass of the peptides varied between 1737.86 and 2758.11; therefore, the final molarity of the peptides varied between 1.8 and 8.6 µM. In experiment 2, the peptides used were 16-mer overlapping by 12 amino acids supplied by Thermo Scientific at 1 mg scale (PEPotec Grade 1) and a concentration of 2.5 mg/mL. Peptide pools contained 19–27 peptides added to the cells at a final concentration of 6.25 µg/mL for each peptide (see Data S4 for a schematic of peptide pools coverage along the viral proteins). Cells were lysed by incubating for 5 minutes in water and then washed with PBS. Biotinylated anti-porcine IFNγ (P2C11), followed by streptavidin-conjugated to alkaline phosphatase, and then, AP Conjugate Substrate Kit (Bio Rad, Kidlington, UK.) was used to visualize spots, which were then counted using an ImmunoSpot Series 6 Ultra-V Analyzer (CTL-Europe, Bonn, Germany). The number of spots was converted into the number of spots per million cells, and the mean of duplicate wells was plotted. In experiments where the number of IFNγ-secreting cells was measured over time, the response to the background (the highest of media/mock/DMSO) was subtracted from the response to whole virus or peptide—this is indicated in the figure legends.

### Fixed-cell assays

#### Immunoperoxidase assay (IPA)

Anti-ASFV antibody titers were determined using an immunoperoxidase assay, by incubating 2-fold serial dilutions of sera on genotype I Ba71V-infected Vero cells, or MA104 clone 1 cells infected with genotype II Georgia 2007/1 that were fixed 16 hours post-infection with 4% paraformaldehyde. Cells were permeabilized with 0.2% Triton X-100, blocked with 5% milk in PBS-0.05% Tween 20 for 1 hour, then incubated with diluted sera for another hour, and finally with HRP-protein A conjugate (Thermo Fisher, Hemel Hempstead,UK). Cells were washed five times with PBS 0.05% Tween20 between each step. Positive wells were identified by AEC staining (2 mM 3-amino-9-ethylcarbazole and 0.015% H_2_O_2_ from Sigma -Aldrich, Gillingham, UK, diluted in 50 mM sodium acetate buffer). All pigs showed non-specific background staining of uninfected cells after incubating with day 0 sera at dilutions varying from 1:16 to 1:128.

### Indirect ELISA

pB602L, pCP204L, and pE183L indirect ELISAs were carried out using *E. coli* expressed recombinant proteins as previously described (24, 30, 54).

### Statistical analysis

Statistical analysis was performed with GraphPad Prism 9 (GraphPad Software, San Diego, USA). Unless stated otherwise, two-way repeated measures analysis of variance (RM-ANOVA) with the Geisser–Greenhouse correction and Tukey’s multiple comparison test was used to compare data between and within groups of animals. Raw data are provided in Table S1.

## Data Availability

The authors confirm that the data supporting the findings of this study are available within the article [and/or] its supplementary materials.

## SUPPLEMENTAL MATERIAL

Supplemental material jvi.00622-24-s0001.pdf Fig. S1-S7 and supporting methods.

Table S1 jvi.00622-24-s0002.xlsx Raw data for all figures.

ASM does not own the copyrights to Supplemental Material that may be linked to, or accessed through, an article. The authors have granted ASM a non-exclusive, world-wide license to publish the Supplemental Material files. Please contact the corresponding author directly for reuse.
