# Supplementary material for: Six adenoviral vectored African swine fever virus genes protect against fatal disease caused by genotype I challenge
Source: J Virol. 2024 Jul 2;98(7):e00622-24. doi: 10.1128/jvi.00622-24 (PMC11264932; doi:10.1128/jvi.00622-24)
Supplement: Supplemental material — Fig. S1-S7 and supporting methods. [file jvi.00622-24-s0001.pdf]

Six adenoviral vectored African swine fever virus genes protect against fatal disease caused by genotype I challenge

Raquel Portugal, Hannah Goldswain, Rebecca Moore, Matthew Tully, Katie Harris, Amanda Corla, John Flannery†, Linda K Dixon, Christopher L Netherton#.

Supplementary Data

Antibodies

Target	Clone/Name	Species	Reference
E183L	RB7	Rabbit	[1]
B602L	CC1	Mouse	[2]
ASFV	AW21	Pig C6	[3]

Protein expression analysis

Vero cells or porcine bone marrow cultures were infected with 100 IU/cell of each rAd. Cells were fixed with 4% paraformaldehyde for 30 minutes or lysed in sample preparation buffer 20 hour post infection (hpi) with rAd. Cells for immunofluorescence were permeabilised with 0.2% Triton X-100 in PBS, incubated with blocking buffer (50 mM Tris pH 7.4, 150 mM NaCl, 0.2% (w/v) gelatin, 10% (v/v) normal goat serum). Primary and secondary antibodies were also diluted in blocking buffer, cells were washed with PBS between all stages. Protein lysates were run down 10% bis-tris gels, transferred to PVDF membranes, blocked with 5% milk powder and then probed overnight with primary antibody diluted in 5% BSA. Secondary antibodies were diluted in 5% milk powder, all solutions were based on TBS containing 0.2% Tween 20. Bands were detected by enhanced chemiluminescence (Pierce) with a Syngene G-box.

References

1. **Goatley L, Reis A, Portugal R, Goldswain H, Shimmon G, Hargreaves Z, et al.** A Pool of Eight Virally Vectored African Swine Fever Antigens Protect Pigs Against Fatal Disease. Vaccines. 2020;8(2):234. 10.3390/vaccines8020234
2. **Cobbold C, Windsor M, Wileman T.** A virally encoded chaperone specialized for folding of the major capsid protein of African swine fever virus. J Virol. 2001;75(16):7221-9.
3. **Rathakrishnan A, Connell S, Petrovan V, Moffat K, Goatley LC, Jabbar T, et al.** Differential Effect of Deleting Members of African Swine Fever Virus Multigene Families 360 and 505 from the Genotype II Georgia 2007/1 Isolate on Virus Replication, Virulence, and Induction of Protection. J Virol. 2022;96(6):e0189921. 10.1128/jvi.01899-21

A

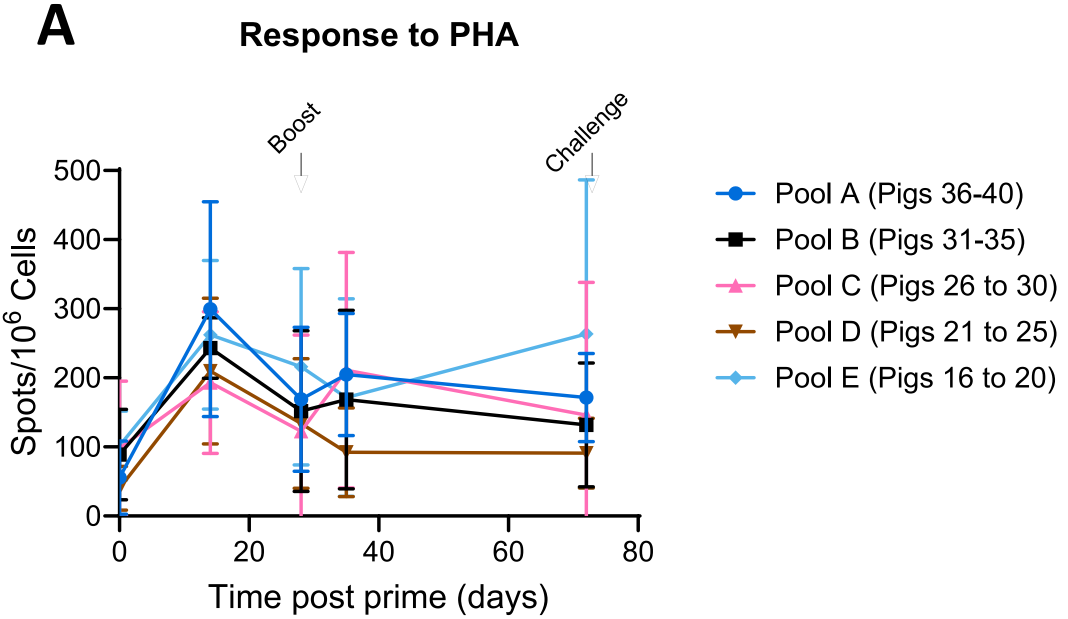

B

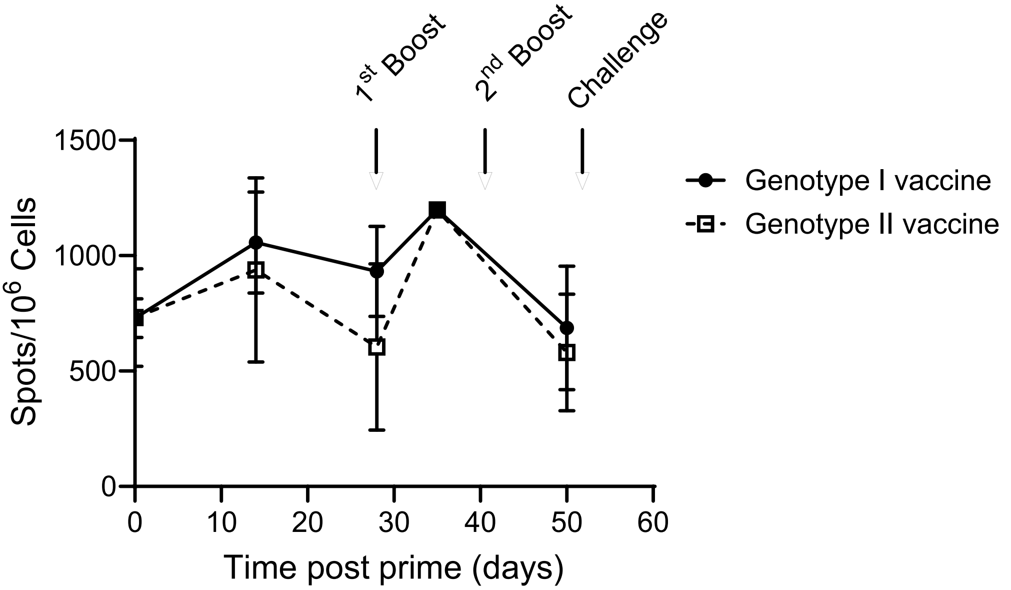

Fig S1: Response of PBMCs to control stimulation with phytohemagglutinin (PHA) in Experiment 1 (A) and experiment 2 (B). In experiment 1 groups of five pigs were immunised with Antigen Pools A, B, C, D, E, boosted on four weeks later (Day 28) and challenged with ASFV 6 weeks after the boost (Day 73). In experiment 2, groups of five pigs were immunised with Antigen Pool B either tailored for genotype I or genotype II ASFV (solid lines and dashed lines respectively) and boosted four and six weeks later (Days 28 and 41 post prime). Blood samples were collected on the indicated times after the first immunisation and cellular response were assessed by interferon γ ELIspot after incubating PBMCs with 2.5 µg/mL phytohemagglutinin (PHA) (A) or 20 µg/mL (B) as a positive control. Spots numbers at day 35 in experiment 2 were above the detection limit and therefore set to 1200 spots. Data indicate the mean of each group and error bars the standard deviation from that mean.

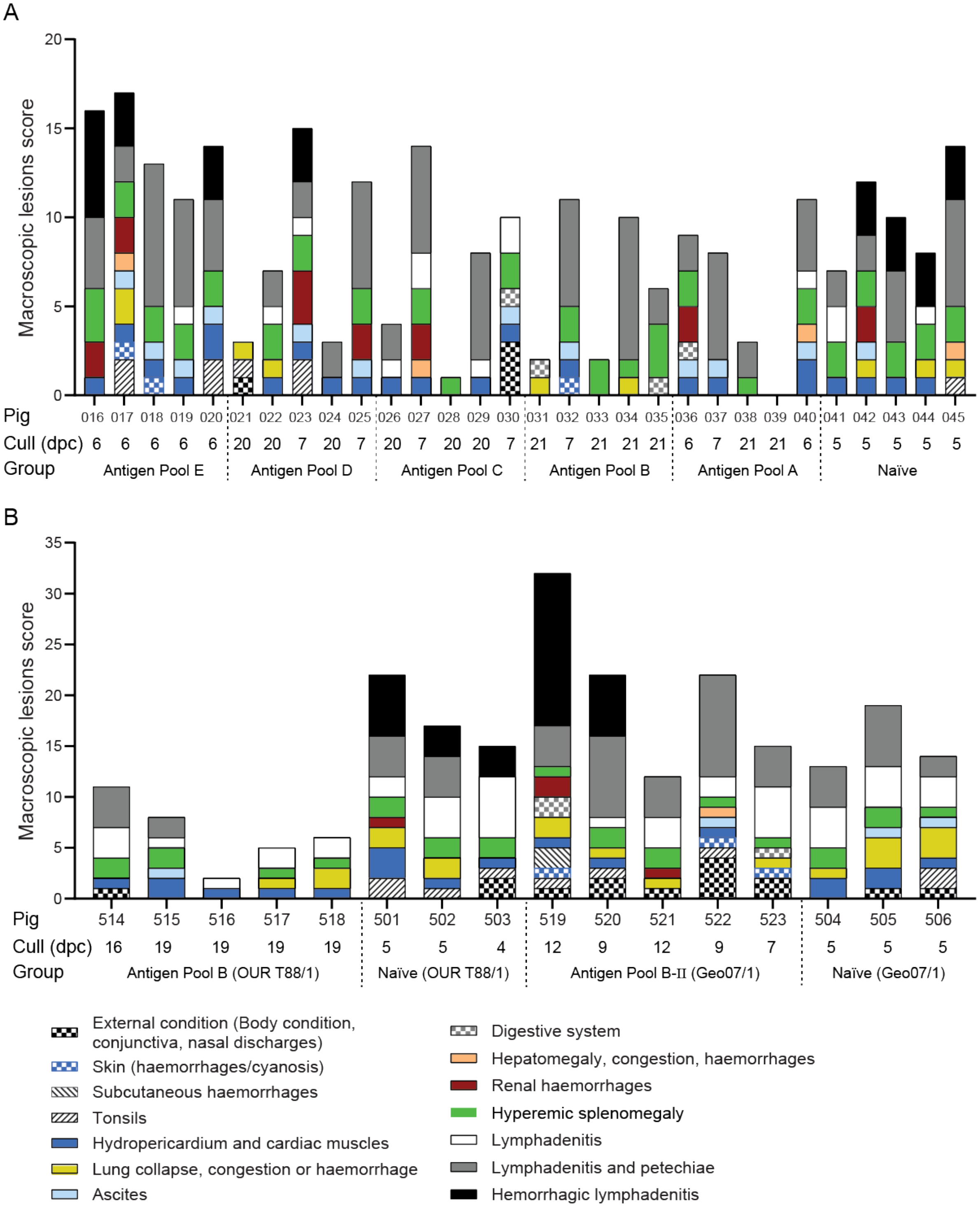

Fig S2: Scoring of macroscopic lesions. Tissues were evaluated and the gross lesions observed *post mortem* from Experiments 1 (A) and Experiment 2 (B) are indicated on the graph by different patterns and/or colours. The animal number of each pig evaluated and the day after challenge that it was euthanised and its experimental group are indicated on the x-axis. For Experiment 2 the challenge virus is indicated after each Group in parentheses.

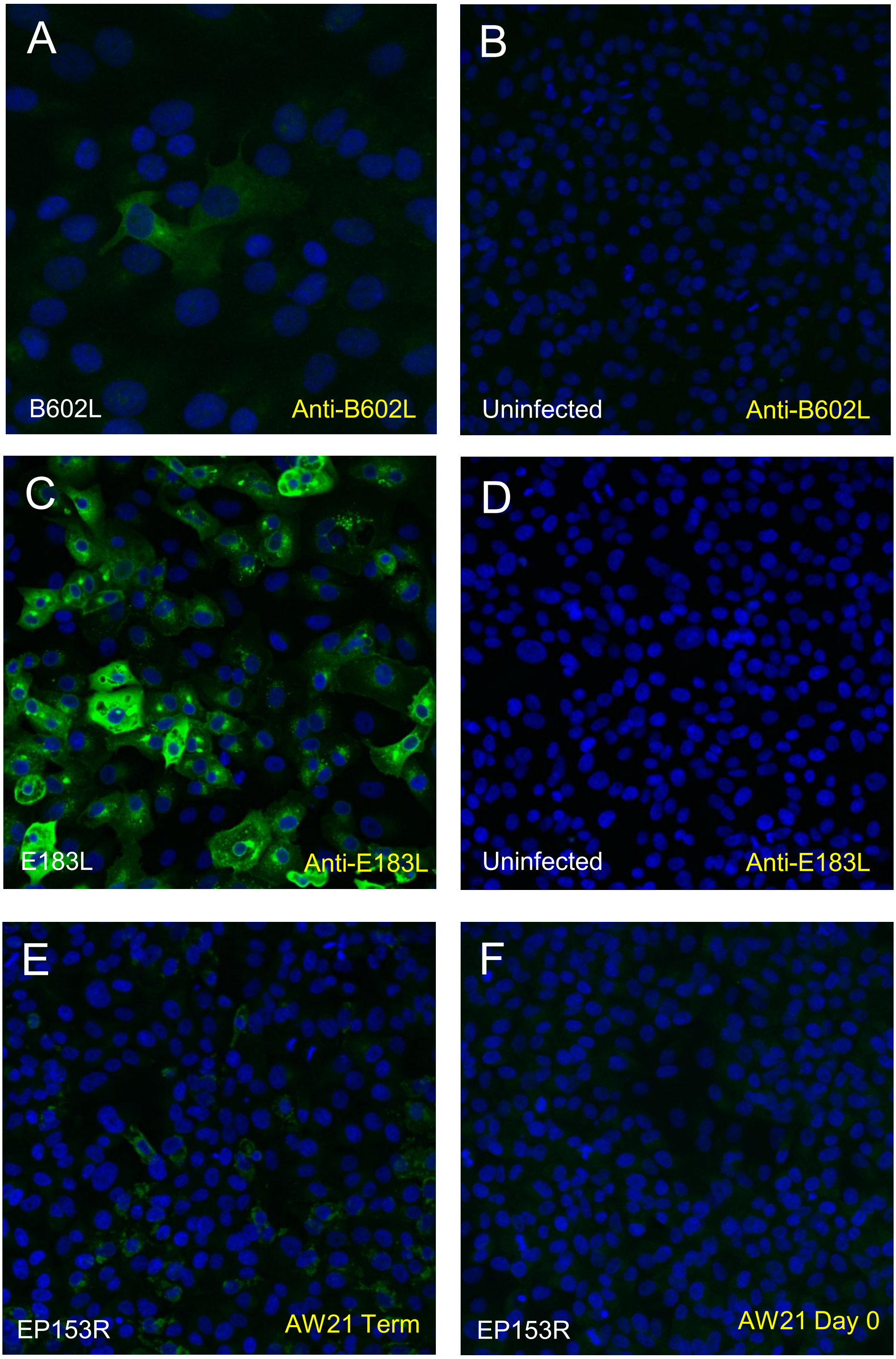

Fig S3: Adenovirus driven expression of ASFV genes. Cells were transduced with replication deficient human adenovirus 5 expressing the indicated genotype II ASFV genes or left uninfected. A. Cells were fixed 24 hours after infection and gene expression was determined by indirect immunofluorescence using anti-B602L rabbit serum CC1 (A,B) anti-E183L rabbit serum RB7 (C, D), or serum taken at termination from a pig that recovered from Georgia 2007/1 (E) or pre-immunisation serum from the same animal (F).

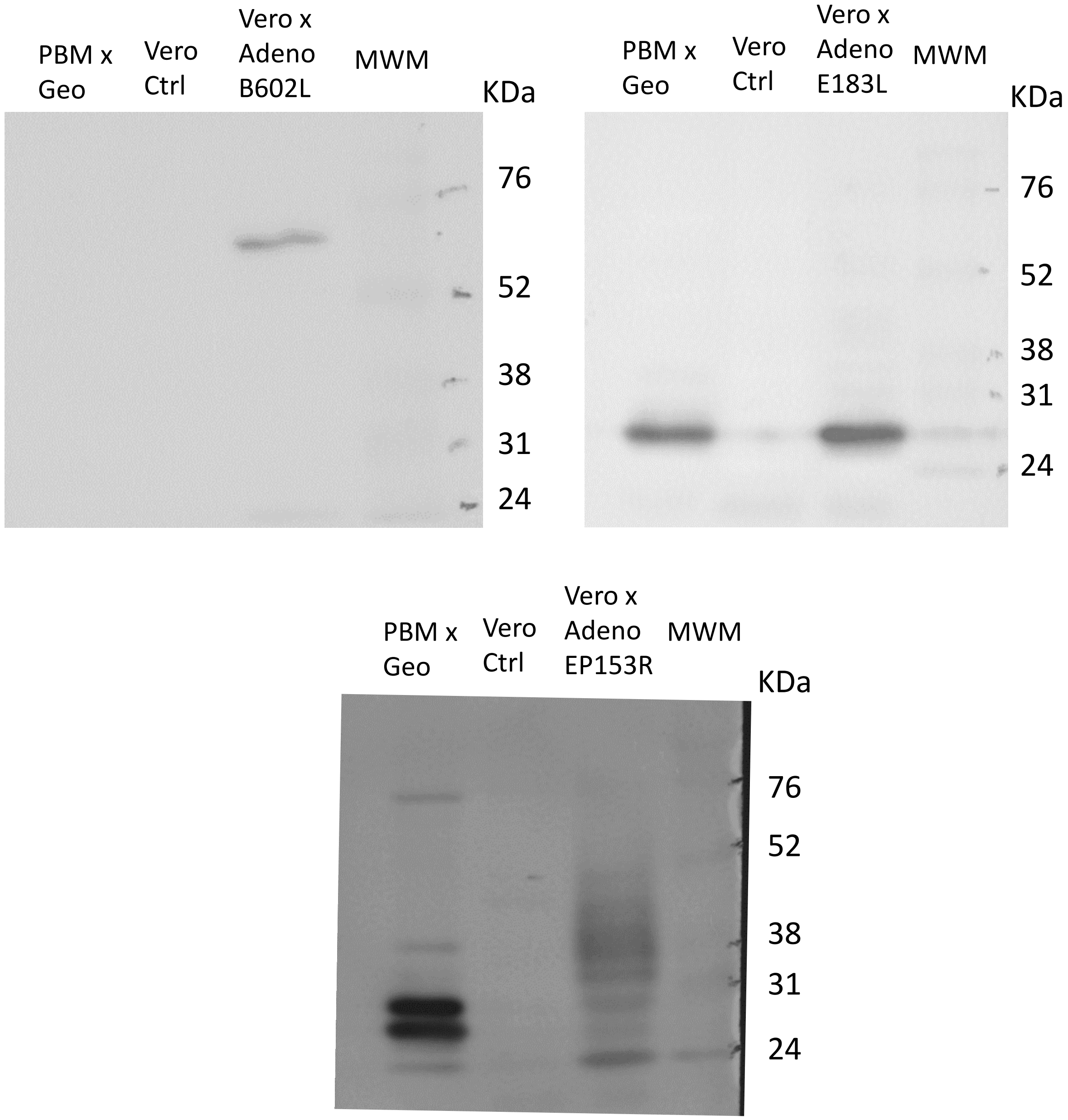

Fig S4: Adenovirus driven expression of ASFV genes. Cells were transduced with replication deficient human adenovirus 5 expressing the indicated genotype II ASFV genes or left uninfected. Cell lysates were collected 24h after infection and viral proteins were detected in immunoblots using the same primary antibodies or sera as in A. Secondary antibodies were anti-rabbit or anti-pig IgG conjugated with HRP. Cellular lysates from Georgia 2007/1 infected porcine bone marrow culture (PBM x Geo) and from uninfected Vero cell culture (Vero Ctrl) were used in the immunoblots as controls. MWM: molecular weight marker.

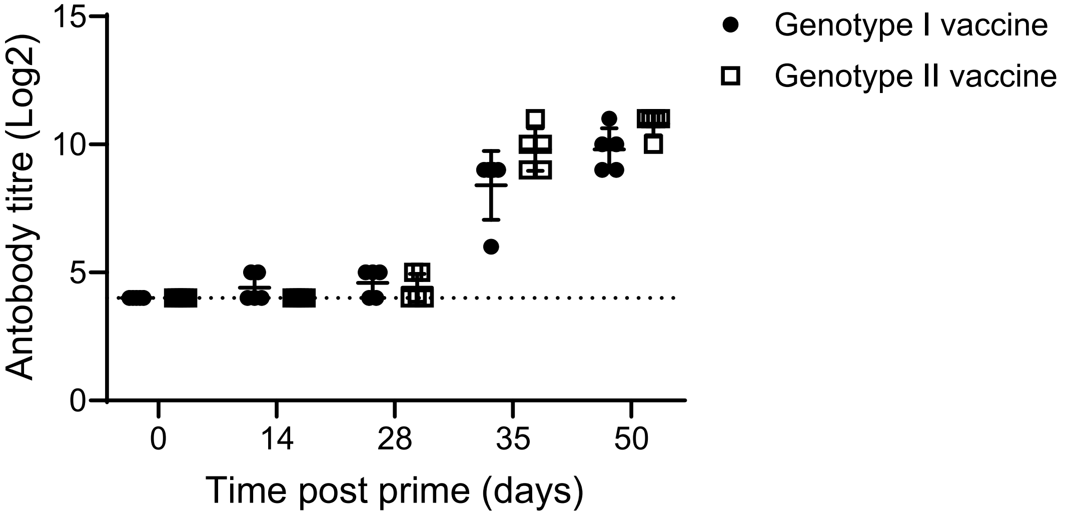

Fig S5: Genotype II ASFV-specific antibody titres in Experiment 2. Antibody responses were determined by immunoperoxidase assay on Georgia2007/1 infected MA104 cells. Groups of five pigs were immunised with Antigen Pool B either tailored for genotype I or genotype II ASFV (Genotype I and II vaccine respectively) and boosted four and six weeks later (Days 28 and 41 post prime). Serum samples were collected from the pigs on the indicated days for antibody titration. Data show the individual values for each pig in the groups and bars indicate the mean and standard deviation of that group. Dotted lines indicate the limits of detection of the assay.

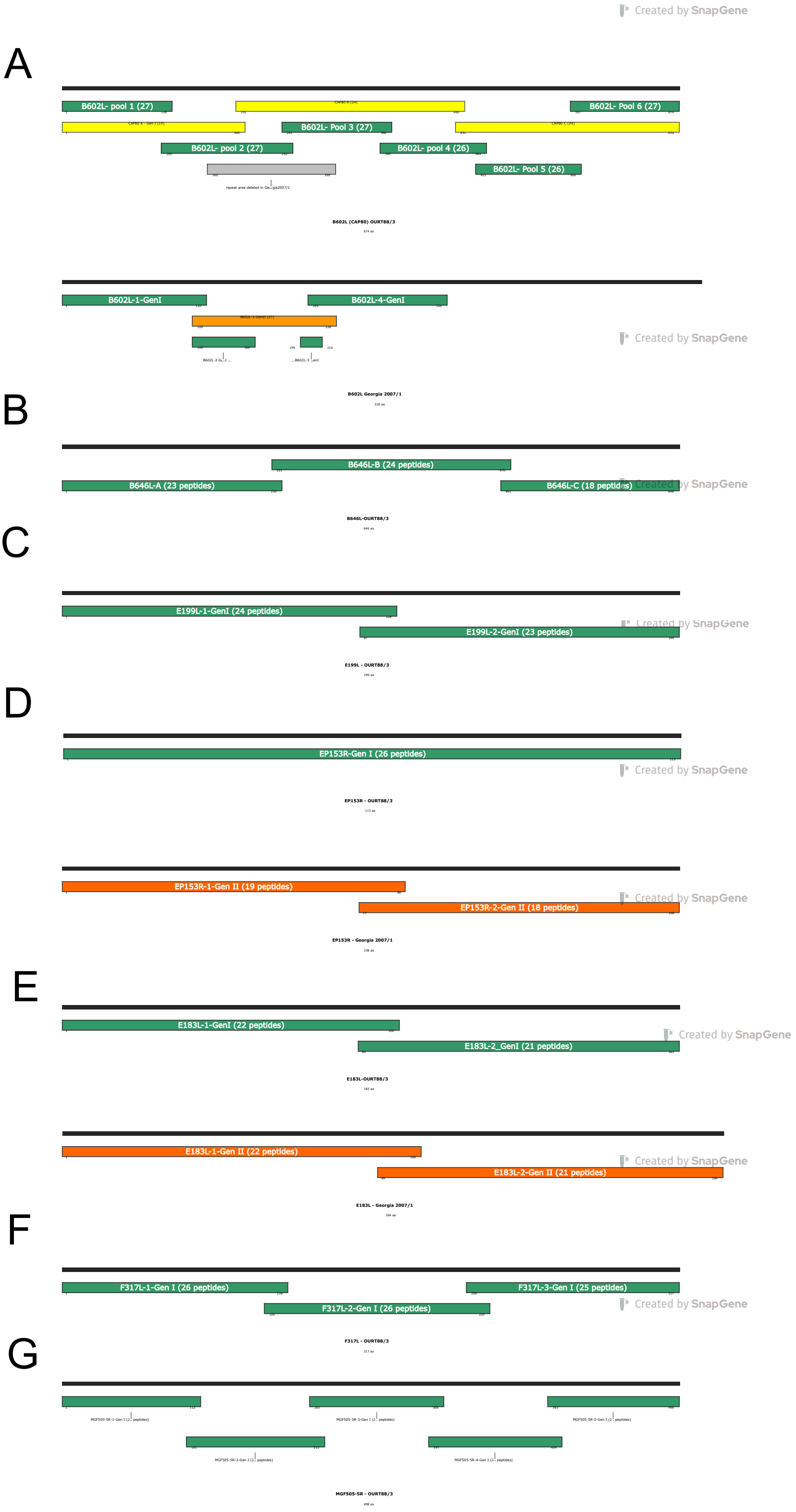

Figure S6: Schematics showing peptide pools used for ELISpot assays to detect antigen specific immune responses to B602L (A), B646L (B), E199L (C), EP153R (D), E183L (E), F317L (F) and MGF505-5R (G). Pools shown in green are those corresponding to the genotype I orthologue of the protein and those in orange are for the genotype II orthologue. Note that most of genotype I and genotype II B602L are identical and therefore only a single pool of genotype II peptides was required to generate pools of the peptide that covered genotype II B602L. Responses to the rest of B602L was tested using the same peptides used to measure responses to genotype I B602L.

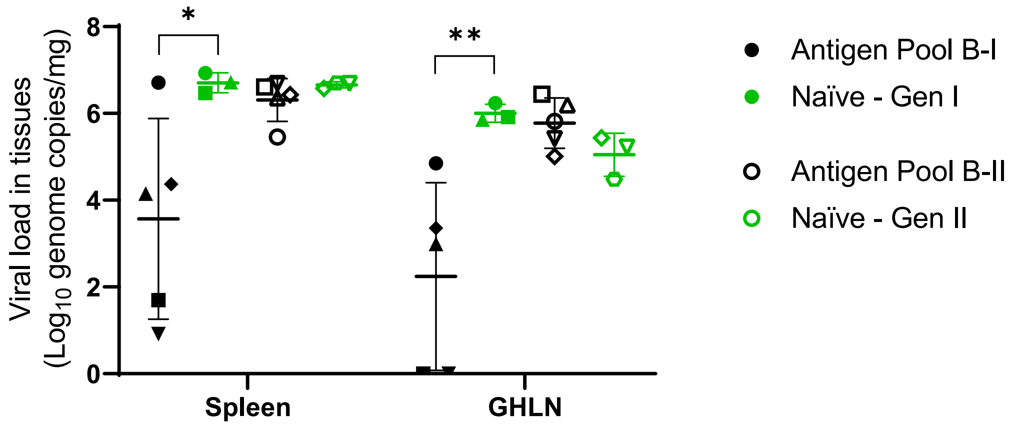

Figure S7: Experiment 2 virological data. Groups of pigs immunised with genotype I Antigen Pool B (solid symbols) or genotype II Antigen Pool B (open symbols), or naïve animals (green symbols) were challenged with OUR T88/1 (solid symbols) or Georgia 2007/1 (open symbols). Viral loads in the spleen and gastro-hepatic lymph node (GHLN) collected post-mortem were determined by qPCR in the individual pigs. Horizontal bars indicate the mean of each group and error bars indicate the standard deviation from that mean. Statistical differences between the naïve and immunised groups as determined by one way ANOVA are indicated by asterisks (* $p<0.05$, ** $p<0.01$).
